# Phosphorylation of CAP1 regulates lung cancer proliferation, migration, and invasion

**DOI:** 10.1007/s00432-021-03819-9

**Published:** 2021-10-12

**Authors:** Jie Zeng, Xuan Li, Long Liang, Hongxia Duan, Shuanshuan Xie, Changhui Wang

**Affiliations:** Department of Respiratory Medicine, Shanghai Tenth People’s Hospital, Tongji University School of Medicine, No.301, Mid Yanchang Rd, Shanghai, 200072 People’s Republic of China

**Keywords:** Adenylate cyclase-associated protein, Phosphorylation, NSCLC, EMT

## Abstract

**Purpose:**

Cyclase-associated protein 1 (CAP1) is a ubiquitous protein which regulates actin dynamics. Previous studies have shown that S308 and S310 are the two major phosphorylated sites in human CAP1. In the present study, we aimed to investigate the role of CAP1 phosphorylation in lung cancer.

**Methods:**

Massive bioinformatics analysis was applied to determine CAP1’s role in different cancers and especially in lung cancer. Lung cancer patients’ serum and tissue were collected and analyzed in consideration of clinical background. CAP1 shRNA-lentivirus and siRNA were applied to CAP1 gene knockdown, and plasmids were constructed for CAP1 phosphorylation and de-phosphorylation. Microarray analysis was used for CAP1-associated difference analysis. Both in vitro and in vivo experiments were performed to investigate the roles of CAP1 phosphorylation and de-phosphorylation in lung cancer A549 cells.

**Results:**

CAP1 is a kind of cancer-related protein. Its mRNA was overexpressed in most types of cancer tissues when compared with normal tissues. CAP1 high expression correlated with poor prognosis. Our results showed that serum CAP1 protein concentrations were significantly upregulated in non-small cell lung cancer (NSCLC) patients when compared with the healthy control group, higher serum CAP1 protein concentration correlated with shorter overall survival (OS) in NSCLC patients, and higher pCAP1 and CAP1 protein level were observed in lung cancer patients’ tumor tissue compared with adjacent normal tissue. Knockdown CAP1 in A549 cells can inhibit proliferation and migration, and the effect is validated in H1975 cells. It can also lead to an increase ratio of F-actin/G-actin. In addition, phosphorylated S308 and S310 in CAP1 promoted lung cancer cell proliferation, migration, and metastasis both in vitro and in vivo. When de-phosphorylated, these two sites in CAP1 showed the opposite effect. Phosphorylation of CAP1 can promote epithelial–mesenchymal transition (EMT).

**Conclusion:**

These findings indicated that CAP1 phosphorylation can promote lung cancer proliferation, migration, and invasion. Phosphorylation sites of CAP1 might be a novel target for lung cancer treatment.

**Supplementary Information:**

The online version contains supplementary material available at 10.1007/s00432-021-03819-9.

## Introduction

Global statistical data show that of newly diagnosed cancer cases, lung cancer is the most common and that it is the leading cause of death among the 36 cancers in the world (Bray et al. 2018). According to American Cancer Society’s 2021 prediction, 235,760 new lung cancers will be reported, which represents about 12.42% of all new cancer cases. The estimated number of deaths from lung cancer will up to 131,880 in 2021 in the United States alone (Siegel et al. 2021). Invasion and metastasis are the main reasons for lung cancer treatment failure and shorter survival time (Kim et al. 2020). Hence, it is necessary to investigate the underlining mechanism of lung cancer metastasis. CAP1 is a cancer-associated protein, and according to UniProt and GO-Cellular component annotation, human CAP1 has an extensive expression. It is well known as a cytoskeleton protein, but it has also been observed to be expressed in the membrane and can secrete to the extracellular region (Hasan and Zhou 2019). High expression of CAP1 can be related to poor prognosis in NSCLC patients and cancer metastasis (Tan et al. 2013; Zeng et al. 2018). A previous study showed that CAP1 can be phosphorylated, of which two phosphorylation sites are related to human beings and act in tandem (Zhang and Zhou 2016). Protein phosphorylation and de-phosphorylation are important signal pathways regulating the process of life (Humphrey et al. 2015; Hunter. 2012). In this study, we applied different databases to analyze CAP1 in various aspects such as expression, prognosis, gene association, and protein–protein-interaction (PPI) network. Human samples were collected to validate the database results. Additionally, we investigated the role of CAP1 phosphorylation in lung cancer A549 cells and validated CAP1’s role in H1975 cells. Both in vitro and in vivo experiments showed that S308/S310 phosphorylation in CAP1 facilitated proliferation and metastasis of lung cancer cells. In addition, we found that phosphorylation of CAP1 promoted EMT, thereby facilitating lung cancer cells’ metastasis.

## Materials and methods

### Bioinformatics analysis

The transcription level of CAP1 in various cancers was analyzed by Oncomine database (https://www.oncomine.org/resource/main.html) (Rhodes et al. 2004). The threshold was set as: *p *value < 1E-4, fold change > 2, and top 10% gene bank. Prognosis analysis was performed by Kaplan–Meier Plotter (http://kmplot.com/analysis/index.php?p=background) (Nagy et al. 2021) and GEPIA (http://gepia.cancer-pku.cn/about.html) (Tang et al. 2017). The UALCAN website (http://ualcan.path.uab.edu/index.html) was used for CAP1 protein expression comparison (Chandrashekar et al. 2017). cBioPortal was used for co-expression analysis (https://www.cbioportal.org/) (Cerami et al. 2012). PPI network and corresponding GO analysis was performed using STRING (https://string-db.org/) (Szklarczyk et al. 2015).

### Human specimens

All clinic specimens were collected in Shanghai Tenth People’s Hospital, Tongji University (China). All blood samples (78 from tumor patients and 62 from a healthy control group) were collected in serum separation tube and then preserved in a refrigerator at 4 °C to protect the proteins and RNA from degradation. A centrifuge was used to spin the samples for 15 min at 3000 rpm at 4 °C within 2 h of initial sample collection. All surgical tissue samples were preserved in liquid nitrogen immediately to prevent protein and RNA degradation. Shanghai Tenth People’s hospital ethics committee approved this study and written informed consent was obtained from all patients.

### Cell culture, plasmids, and transfection

Human lung cancer cell line A549 and H1975 were purchased from the FDCC (Shanghai, China). A549 and H1975 cells were grown in RPMI 1640 medium (Thermo, Suzhou, China) supplemented with 10% FBS (fetal bovine serum) (Gemini, USA) and 1% penicillin/streptomycin (Gemini, USA). A549 cells were transfected with CAP1 shRNA-lentivirus (Lingke Co. China) according to the manufacturer’s instructions. CAP1 wild type (hereafter referred to as WT), S308A/S310A (mimicking a constitutively non-phosphorylated site, hereafter referred to as AA), and S308D/S310D (mimicking a constitutively phosphorylated site, hereafter referred to as DD) mutant plasmids were constructed with pcDNA4 plasmid. For the rescue experiment, lentivirus CAP1 knockdown A549 cells (sh-A549) were seeded in 6-well plates and were co-transfected with 2000 ng plasmids by Lipofectamine 3000 (Invitrogen, USA), according to the manufacturer’s instructions.

### Lentivirus and siRNA infection

Lentiviral vectors for the human CAP1-expressing sequence were constructed by Lingke Co. (Shanghai, China). The target sequences for sh-RNA-1 and sh-RNA-2 were [5-CACGACATTGCAAATCAAG-3] and [5-AGATGTGGATAAGAAGCAT-3], respectively. CAP1 shRNA-lentivirus and negative control lentivirus were prepared and titered to 10^9^ TU/ml (transfection unit). All viruses were fused with green fluorescent protein (GFP). About 30,000 A549 cells in 100 μl were seeded in a 24-well plate. When they reached 50–70% confluence, lentivirus and polybrene were added according to the manufacturer’s instructions. After co-culture for 12–16 h, the medium was refreshed. 72 h after transfection, the transfection efficiency was observed with a fluorescence microscope. CAP1 siRNAs were constructed by Sangon Biotech (Shanghai, China). S1 and S2 sequences are (F) 5′-GAACCGAGGCAGCAAGUUUUU-3′, (R) 5′-AAACUUGCUGCCUCGGUUCUU-3′; (F) 5′-AAAGCAUGGCAGCCAUCUGUU-3′, and (R) 5′-AAAACAGAUGGCUGCCAUGCU-3′, respectively. H1975 cells were seeded in the 6-well plate and cultured overnight. When confluence reached 50%, CAP1 siRNA were transfected into cells by Lipofectamine 2000 (Invitrogen) according to the manufacturer’s instructions.

### Microarray analysis

The Agilent SurePrint G3 Human Gene Expression v3 Microarray (8*60K, Design ID:072363) was used in this experiment to perform data analysis on 15 samples. NanoDrop ND-2000 (Thermo Scientific) was used to quantify total RNA and the Agilent Bioanalyzer 2100 (Agilent Technologies) was used to assess the RNA integrity. Total RNA was transcribed into double-stranded cDNA, synthesized into cRNA, labeled with Cyanine-3-CTP, and hybridized onto the microarray. The arrays were scanned by the Agilent Scanner G2505C (Agilent Technologies) after washing. Feature Extraction software (version10.7.1.1, Agilent Technologies) was used to analyze array images to generate raw data. Genespring (version13.1, Agilent Technologies) was used to finish the basic analysis of the raw data. The threshold set for up- and down-regulated genes was a fold change ≥ 2.0 and a *p* value <  = 0.05.

### Cell proliferation assays

The Cell Counting Kit-8 (CCK-8, Yeasen, China) was used for the cell proliferation assay. In each well of 96-well plates, approximately 1,000 cells were seeded. At each detection time point, 10 μl CCK-8 were added into each well, which was then incubated for 2 h at 37 °C in an incubator with 5% CO_2_, following the manufacturer’s instructions. Epoch Microplate Spectrometer (Winooski, BioTek) was used to detect the optical density (OD) at a 450 nM absorbance reading. In the cell clone assay, 1000 cells suspended with 2 ml RPMI 1640 medium (Thermo, Suzhou, China) supplemented with 10% FBS (fetal bovine serum) (Gemini, USA) and 1% penicillin/streptomycin (Gemini, USA) were seeded into 6-well plates. After 10 days, clones were fixed with 95% ethanol for 10 min, stained with 0.1% crystal violet, and washed by ddH_2_O for three times. Next, they were air-dried, photographed, and then analyzed by ImageJ software (National Institutes of Health, USA). Three independent experiments were conducted, and data were analyzed by Student’s *t* test.

### Cell migration and invasion assays

In wound-healing assays, 6-well plates were used to culture cells. A sterile 10 μl pipette tip was used to generate a scratch through each well until cell confluence reached 95%. At each time point, the wound images were captured with a microscope (Olympus, Tokyo, Japan). Relative migration rate was calculated by the following formula: (wound width at 0 h—wound width at *n* hour)/wound width at 0 h. For Transwell assays, cells were harvested and re-suspended in the serum-free medium containing 0.1% bovine serum albumin before seeded onto Transwell chambers. 500 μl RPMI 1640 medium (Thermo, Suzhou, China) supplemented with 10% FBS (Gemini, USA) and 1% antibiotics (penicillin–streptomycin, Gibco, USA) were added to the lower chamber. After 12 h of incubation at 37 °C in an incubator, cells in the upper chamber were removed by cotton tips and washed 3 times with 1 × phosphate buffer saline (PBS). The lower surface of the chamber was fixed with 95% ethanol for 10 min, stained with 0.1% crystal violet solution for 10 min, washed with ddH_2_O for 3 times, air-dried, and photographed in five randomly selected fields for each well under a microscope (Olympus, Tokyo, Japan). The cells in the selected fields were counted by ImageJ software (National Institutes of Health, USA). The experiments were repeated three times, and the results were analyzed with Student’s *t* test.

### Western blot analysis

30 μg of cell protein was used for western blot analysis. Samples were dissolved in 10% SDS-PAGE and transferred to polyvinylidene fluoride (PVDF) membranes (Merck Millipore, Germany). PVDF membranes were activated by methanol before use. The membranes were blocked for 1 h in a solution of 5% fat-free milk powder dissolved in PBS with 0.1% Tween 20 (PBST) followed by incubation with primary polyclonal and monoclonal antibodies (E-Cadherin: 1:1000, Abcam, ab76055; N-Cadherin: 1:1000, Abcam, ab18203; Vimentin 1:1000, Abcam, ab92547; pCAP1:1:500, donated by Prof. Field (Freeman and Field 2000) and the internal control (Gapdh 1:10,000, Abcam, UK; Actin 1:2000, Abcam, Cambridge, USA) overnight at 4 °C. Blots were washed in PBST three times and incubated with corresponding secondary antibodies at room temperature for 1 h, and then washed in PBST three times. LI-COR Odyssey Imaging System (LI-COR Biosciences, Lincoln, USA) was used to process the membrane.

#### RT-PCR

RT-PCR was used to determine the expression of CAP1 mRNA in A549 and H1975 cells. Total RNA of cells was isolated by standard procedures. Primer sequence: sense (5′-3′): 5′-CAAGCCTGGCCCTTATGTGA-3′, antisense (3′-5′):3′-CTCCACTTCTTGCCCTCCAG-5′ was synthesized by Sangon Biotech (Shanghai. China). Then, RT-PCR was performed using the Takara PrimeScript RT reagent kit (Kusatsu, Japan) and FastStart Universal SYBR green kits according to the manufacturer’s instructions.

### Subcutaneous implantation and tail vein mouse model

Animal Care and Use Committee at Tongji University (Shanghai, China) approved the animal experiment. Nude mice were purchased from Shanghai Laboratory Animal Center of China. sh-A549 was separately transfected with CAP1 WT, AA, and DD plasmids. The three transfected types of cells were used for generating xenograft models. For subcutaneous tumor formation assay, 4 × 10^6^ cells in 100 μl PBS were subcutaneously injected into nude mice (8 mice/group). The tumor volume and mice weight were measured every week. Subcutaneous xenografts were viewed by Berthold LB983 small animal live imaging system. All mice were terminated 6 weeks after injection. For the tail vein mouse cancer model, 2 × 10^6^ cells in 100 μl PBS were injected into nude mice through the tail vein (8 mice/group). The survival time of the mice was recorded.

### H&E and immunohistochemical (IHC) assay

Organs were fixed with 4% paraformaldehyde. The organs were embedded, sectioned, dewaxed in xylene, rehydrated in graded ethanol, and stained with H&E using a standard procedure. Neutral gum was used to fix the sections, and images were captured using a light microscope (Olympus, Tokyo, Japan). Subcutaneous tumor sections were deparaffinized and rehydrated with the standard protocol. Antigen retrieval was performed by citrate method. Slides were immersed in boiled 10 mM sodium citrate buffer pH6.0 for 20 min, and then, slides were cooled on a bench for 30 min. After that slides were immersed in PBS until no bubbles were present. IHC staining was performed with antibodies specific for E-Cadherin (1: 100, Abcam, ab76055), N-Cadherin (1:300, Abcam, ab18203), and Vimentin (1:300, Abcam, ab92547). Immunohistochemically stained tissue sections were assessed separately by at least two pathologists.

### Statistical analysis

SPSS version 20.0 was used for statistical analysis. The mean ± standard error (SE) for at least three independent experiments was determined. Student’s *t *test, Chi-square test, and one-way ANOVA were used to analyze the experiment results. *p* < 0.05 was recognized as significant.

## Results

### The transcription level of CAP1 in different cancers and its role in NSCLC

**Fig. 1:**
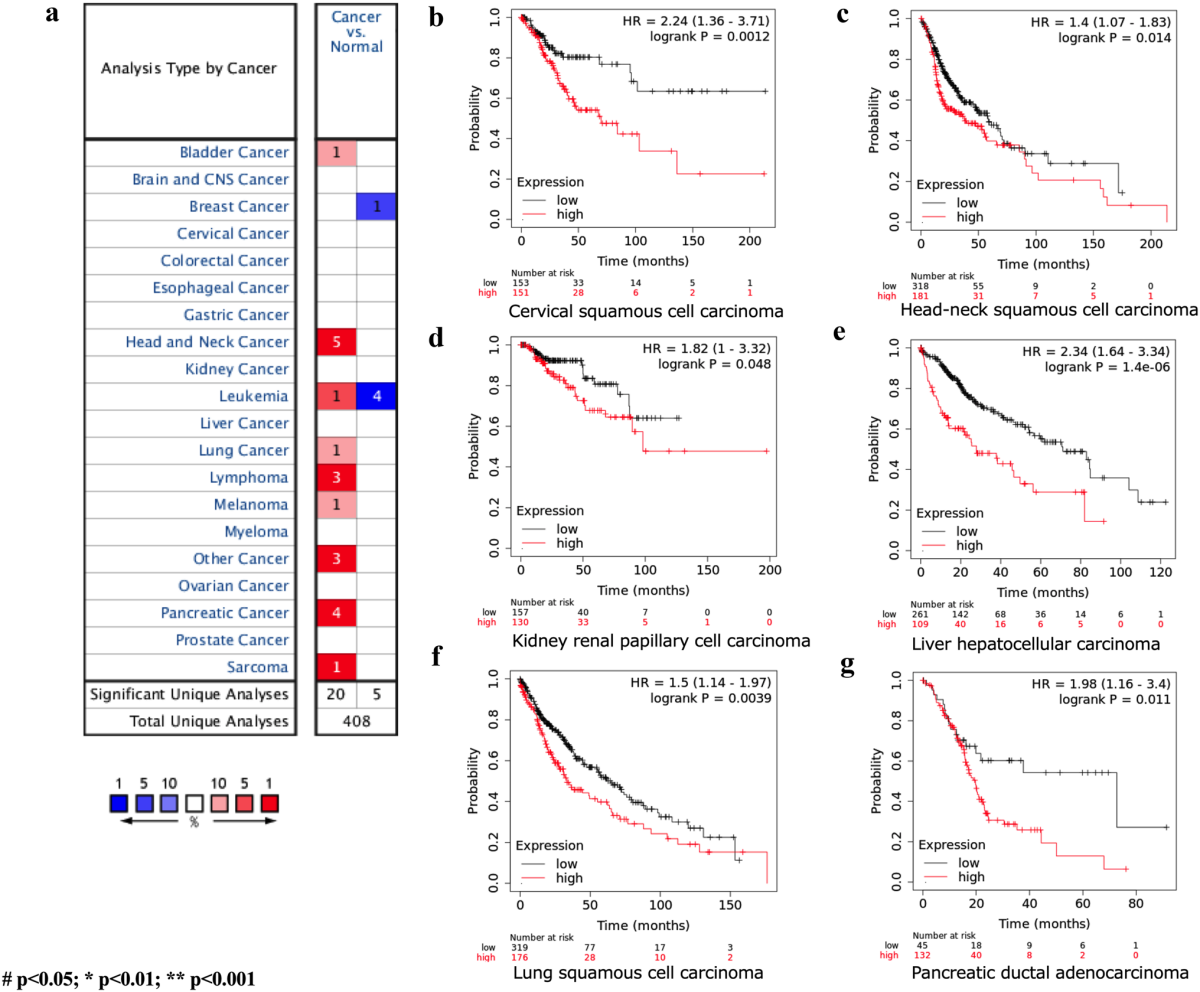
The transcription level of CAP1 in different cancers and the correlation of CAP1 expression level and overall survival. **a** Compared CAP1 transcription level between cancer and normal tissue from Oncomine database. Red represents higher transcription level and blue represents lower transcription level in tumor tissue when compared with normal tissue. **b**–**g** The survival curve of CAP1 high expression (red) and low (black) expression in different cancers were plotted by Kaplan–Meier Plotter (#*p* < 0.05; *p < 0.01; **p < 0.001)

**Fig. 2:**
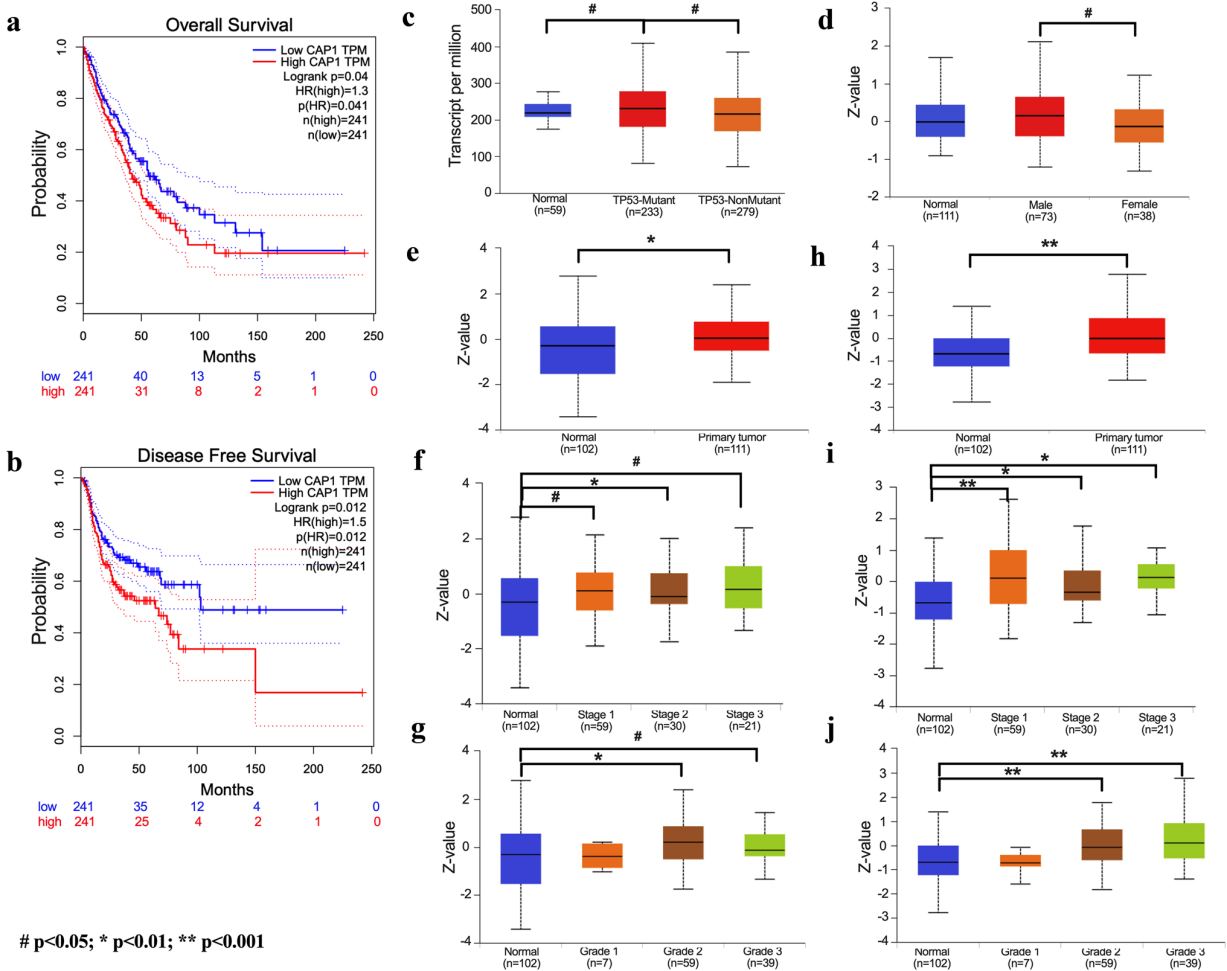
The correlation of CAP1 expression level and survival and detailed protein CAP1 expression level based on various conditions in lung cancer. **a**, **b** Kaplan–Meier curves of OS and DFS. High level of CAP1 expression was associated with poor prognosis. c. CAP1 translation level comparison between normal and TP53-mutant or non-mutant NSCLC patients from TCGA database. d. PhosphoProtein analysis from Clinical Proteomic Tumor Analysis Consortium (CPTAC) showed CAP1 protein expression was higher in male LUAD patients than female LUAD patients. **e**–**j** CAP1 protein phosphorylation rate between normal control and primary LUAD tumor samples in the manner of different stage and grade (**e**–**g** dataset: NP_001099000.1: S255; **h**–**j**, dataset: NP_001099000.1: S275). (#*p* < 0.05; *p < 0.01; **p < 0.001)

CAP1 mRNA transcription level between cancer and normal tissue was analyzed by Oncomine database (Rhodes et al. 2004). A total of 171 datasets, which included 31,692 samples, were selected, and the threshold was set as: *p *value < 1E-4, fold change > 2 and top 10% gene bank. Results showed that 108 datasets met the threshold, and among them, 20 analyses showed higher CAP1 mRNA level in cancer tissue when compared with normal tissue. Five analyses showed lower CAP1 mRNA level in cancer tissue. CAP1 was overexpressed in most cancers (Fig. 1a), including bladder, head and neck, leukemia, lung, lymphoma, melanoma, pancreatic, and sarcoma cancer. The overall survival (OS) between CAP1-high-expression and CAP1-low-expression mRNA level in different cancers was analyzed by Kaplan–Meier Plotter database (Nagy et al. 2021). A total of 7 462 samples were included. It was observed that CAP1-high-expression mRNA level associated with poor prognosis in various cancers, which include cervical squamous cell carcinoma, head–neck squamous cell carcinoma, kidney renal papillary cell carcinoma, liver hepatocellular carcinoma, lung squamous cell carcinoma, and pancreatic ductal adenocarcinoma (Fig. 1b–g). TCGA database from GEPIA was used to validate the OS between CAP1 mRNA expression levels in lung cancer patients and the data revealed that lung cancer patients with higher CAP1 mRNA expression level corresponded to a worse prognosis (Tang et al. 2017). A total of 482 patients were enrolled, and the cutoff-high and cutoff-low were set as 75% and 25%. 241 in high CAP1 expression group and 241 in low CAP1 expression group were included. The *p *value was 0.04, HR (high) was 1.3, and p(HR) was 0.041 (Fig. 2a). The DFS between CAP1-high-expression level and low-expression level in lung cancer patients was also compared in TCGA and data revealed that NSCLC patients with high CAP1 expression level gained a shorter DFS time (Fig. 2b). The *p *value was 0.012, HR (high) was 1.5, and *p* (HR) was 0.012. These results revealed that CAP1-high-expression correlated with poor diagnosis in NSCLC patients. We looked for more information on CAP1 expression in NSCLC from UALCAN website (Chandrashekar et al. 2017). It was observed that TP53-mutated lung adenocarcinoma patients showed higher CAP1 transcript level when compared with normal control and TP53 non-mutant LUAD patients (Fig. 2c). PhosphoProtein analysis from Clinical Proteomic Tumor Analysis Consortium (CPTAC) (Chandrashekar et al. 2017) showed that CAP1 protein expression was higher in male LUAD patients than female LUAD patients (Fig. 2d). When the CAP1 protein phosphorylation rate between normal control and primary LUAD tumor samples was compared, it was observed that there was a higher protein phosphorylation rate in the NSCLC patient (Fig. 2e–g, datasets: NP_001099000.1: S255; Fig. 2h–j NP_001099000.1: S275). Next, we assessed the phosphorylation rate at different stages and grades. Compared with normal healthy control, it was observed that higher stages or grades corresponded to higher CAP1 phosphorylation rates (Fig. 2f, g, i, j).

### Genes correlated with CAP1 and CAP1 protein network in NSCLC

**Fig. 3:**
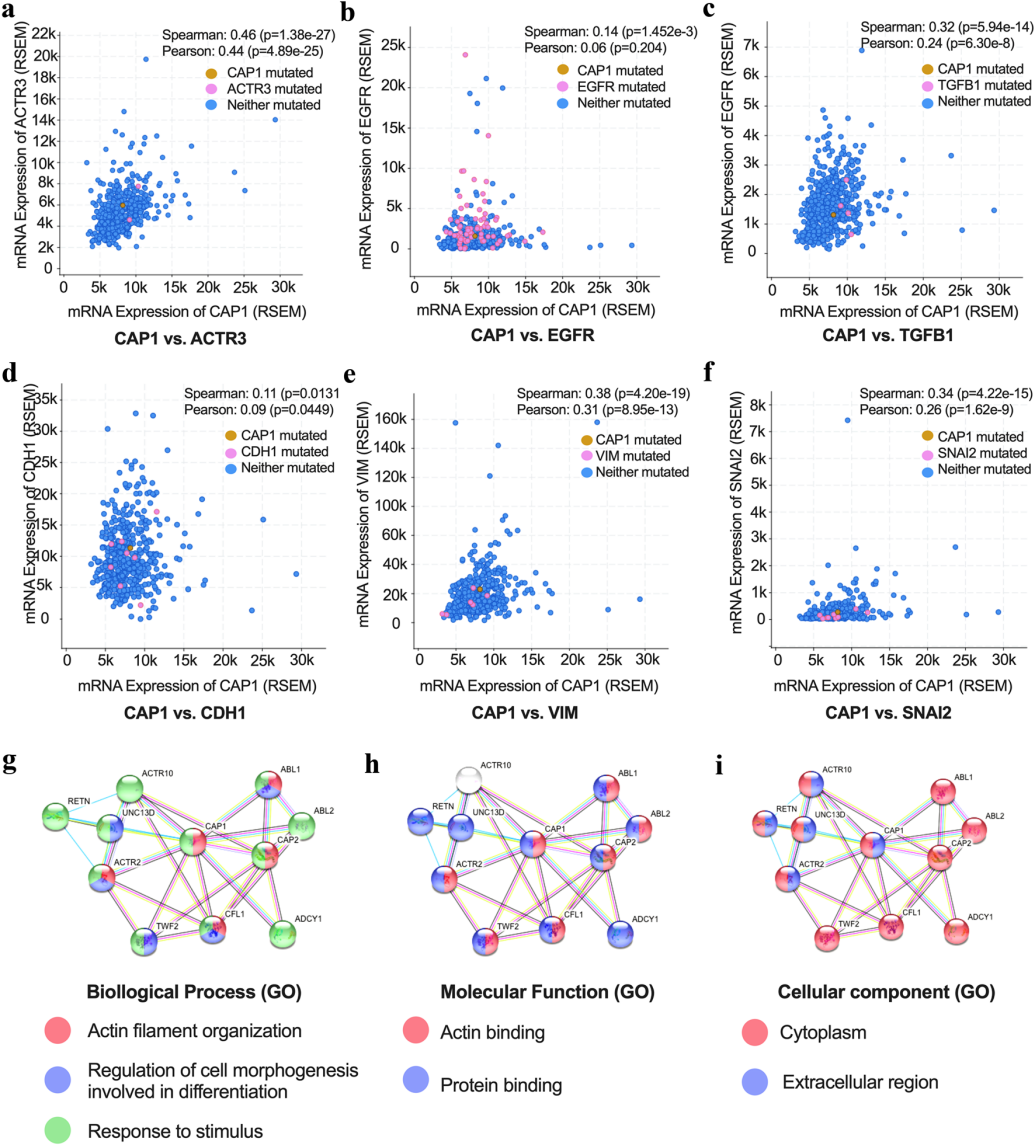
CAP1 co-expression and PPI network analysis in lung cancer. **a**–**f** Summary of selected genes mediating actin skeleton, carcinogenesis, and EMT. **g**–**i** GO analysis of CAP1 PPI network

Analysis from cBioPortal (Cerami et al. 2012) showed CAP1 had a strong correlation with many key genes regulating cytoskeleton, cell adhesion, motility, and migration. CAP1 showed a higher correlation with many actin or actinin genes, including ACTN4, ACTG1, ACTR3, ACTB, ACTR2, ACTN1, ACTA2, and ACTG2. It was detected that CAP1 had a higher correlation with some oncogene-driven genes such as EGFR, ALK, and ROS1. CAP1 also showed higher correlation with EMT genes, which included CDH1, VIM, ZEB1, ZEB2, and TGFB1 (Fig. 3a–f, Table 1). We set p < 0.05 for significance in Spearman’s correlation test. For r value: < 0.10 indicates negligible correlation, between 0.10 and 0.39 indicates weak correlation, between 0.40 and 0.69 indicates moderate correlation, between 0.70 and 0.89 indicates strong correlation, and > 0.9 indicates very strong correlation (Schober et al. 2018). CAP1 PPI network was analyzed by String database (Szklarczyk et al. 2015). The following ten proteins showed a close connection with CAP1: CAP2, ABL1, ABL2, ACTR2, ACTR10, UNC13D, TWF2, CFL1, ADCY1, and RETN. According to Gene Oncology (GO) analysis, the biological process for the CAP1 PPI network is mainly associated with actin, cell morphogenesis and response to stimulus (Fig. 3g). Molecular function is mainly associated with actin and protein binding (Fig. 3h). For the cellular component, CAP1 and associated proteins were mainly present in the cytoplasm and extracellular region (Fig. 3i).

**Table 1:** Genes correlated with CAP1 in NSCLC

Gene symbol	Full name	Function	Spearman’s correlation	*p *value
ACTN4	Actinin alpha 4	Cytoskeletal proteins, actin-binding protein, metastatic processes	0.20245834	3.47698E-06
ACTG1	Actin gamma 1	Cytoskeleton, cell motility	0.179569244	4.01659E-05
ACTR3	Actin related protein 3	Cell shape, motility	0.460179609	1.86E-28
ACTB	Actin beta	Cell motility, structure, integrity, and intercellular signaling	0.414475056	7.01E-23
ACTR2	Actin related protein 2	Cell shape, motility	0.405735999	6.58E-22
ACTN1	Actinin alpha 1	Cytoskeletal proteins, actin-binding protein	0.282947878	5.66E-11
ACTA2	Actin alpha 2	Cell motility, structure, integrity, and intercellular signaling	0.277011179	1.46E-10
ACTG2	Actin gamma 2	Cytoskeleton, cell motility	0.212834039	1.04117E-06
EGFR	Epidermal growth factor receptor	Cell proliferation, migration	0.130587588	0.002931663
RNF11	Ring finger protein 11	Protein–protein interactions	0.448577233	5.83E-27
ALK	ALK receptor tyrosine kinase	Cell proliferation and migration	0.088253524	0.04488461
ROS1	ROS proto-oncogene 1	Cell differentiation, proliferation, growth, and survival	0.17350029	7.32264E-05
MAP2K1	Mitogen-activated protein kinase kinase 1	Cell growth, adhesion, survival, and differentiation	0.279487546	9.87E-11
NRAS	NRAS proto-oncogene	Cell differentiation, proliferation, division ,and movement	0.317476518	1.43E-13
FGFR1	Fibroblast growth factor receptor 1	Cell proliferation, differentiation, and migration	0.115234145	0.008727413
CDH1	Cadherin 1	Cell–cell adhesions, mobility, and proliferation of epithelial cells	0.102859602	0.019318321
VIM	Vimentin	Cytoskeleton, cell attachment, migration, and signaling	0.383564448	1.45E-19
SNAI2	Snail family transcriptional repressor 2	Cell adhesion, proliferation and EMT	0.340518536	1.68E-15
ZEB1	Zinc finger E-box binding homeobox 1	Represses E-cadherin promoter and induces an epithelial-mesenchymal transition (EMT) by recruiting SMARCA4/BRG1	0.271559298	3.43E-10
ZEB2	Zinc finger E-box binding homeobox 2	Represses transcription of E-cadherin	0.305175006	1.32E-12
GRHL2	Grainyhead like transcription factor 2	Tumor suppressor, enhance cell proliferation and suppress apoptosis	− 0.266125305	7.86558E-10
TGFB1	Transforming growth factor beta 1	Cell proliferation, differentiation and growth, promote EMT in cancer cells	0.324786912	3.64E-14
SMAD3	SMAD family member 3	Cell growth and migration	0.156492838	0.00035468

### CAP1 was overexpressed in NSCLC serum samples and correlated with poor clinical outcomes

**Fig. 4:**
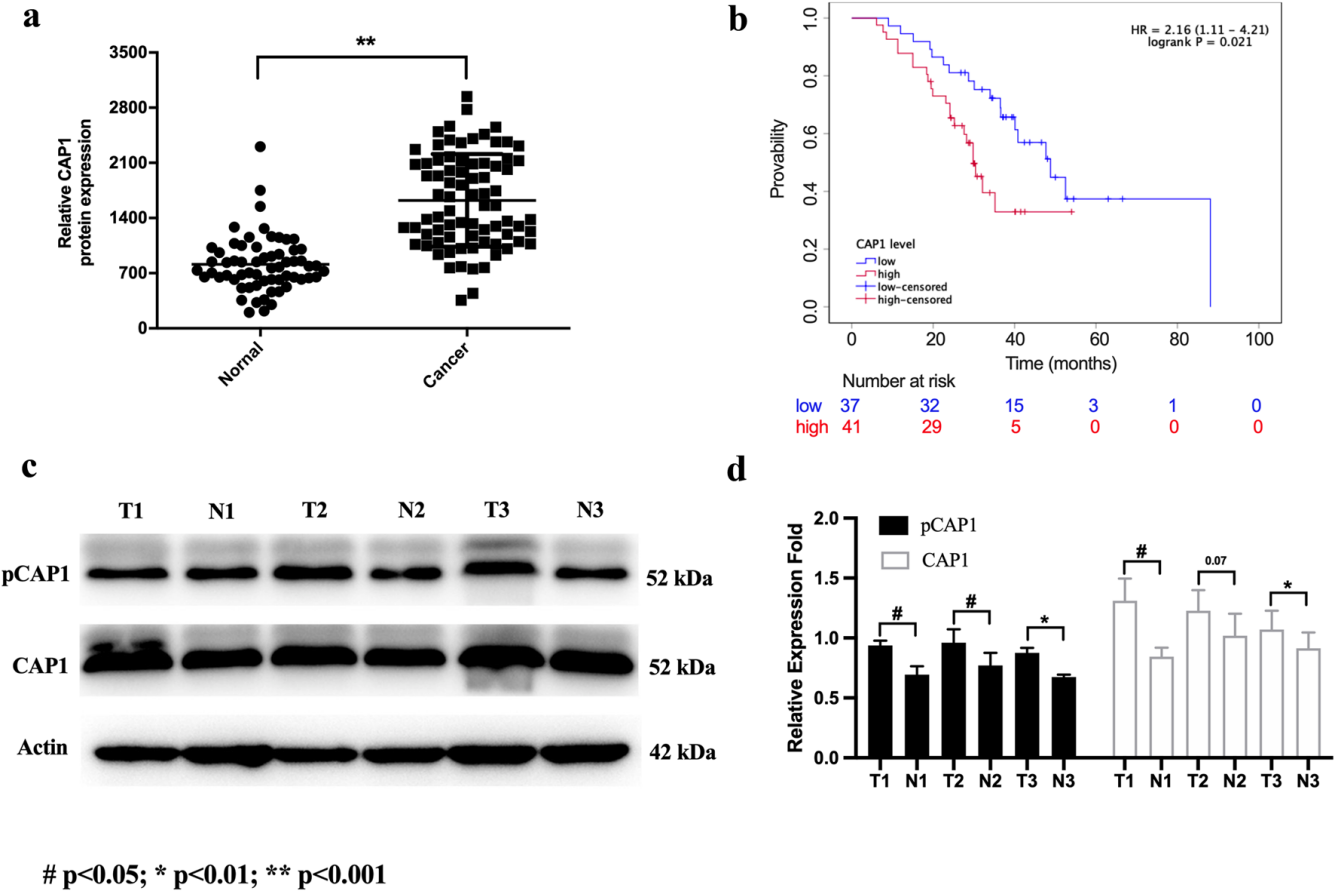
CAP1’s expression level in human samples. **a** The expression of CAP1 was detected in the serum of both cancer patients and normal people by ELISA test (Normal: 62; Cancer: 78). **b** Kaplan–Meier curves of OS in NSCLC patients. High level of serum CAP1 expression was associated with poor prognosis (*p* = 0.021). **c**, **d** Western blot analysis for pCAP1 and CAP1 in human samples and quantified expression level of proteins. (#*p* < 0.05; *p < 0.01; **p < 0.001)

To investigate the expression levels of CAP1 in NSCLC patients and normal people, we analyzed CAP1 protein levels in human serum both from cancer patients and normal people. Results showed that CAP1 protein levels were significantly upregulated in the serum of NSCLC patients compared with that of normal people (*p* < 0.0001, Normal 812.8 ± 44.95, *N* = 62, Cancer 1624 ± 66.47, *N* = 78) (Fig. 4a). Kaplan–Meier survival analysis indicated higher serum CAP1 level associated with shorter OS (*p* = 0.021) (Fig. 4b). The baseline characteristics of the 78 NSCLC patients included in this study are showed in Table 2. Poor differentiation (*p* = 0.0388) and EGFR mutations (*p* = 0.0486) were associated with higher CAP1 expression level. Multivariate analysis results are shown in Table 3. Poor differentiation was associated with poor OS (HR = 2.16, 95% CI 1–4.69, *p* = 0.05). Three paired lung cancer tissue and adjacent normal tissue were collected. It was observed that the pCAP1 and CAP1 protein levels in lung cancer patients’ tumor tissue were higher compared with adjacent normal tissue (Fig. 4c. d). Patient plasma samples were collected between December 2018 and July 2019, and surgical samples were collected between July and August 2021.

**Table 2:** Baseline characters of NSCLC patients

Factors	CAP1 (*n* = 78)		*p *value
	Low (*n* = 37)	High (*n* = 41)	
Age (years)			0.3448
65	22	20	
> 65	15	21	
Gender			0.2197
Male	29	27	
Female	8	14	
Smoking status			0.4693
Nonsmoker	28	28	
Ever-smoker	9	13	
Histologic type			
Adenocarcinoma	27	36	0.0970
Non-Adeno	10	5	
Stage			0.6230
I–II	9	12	
III–IV	28	29	
Differentiation			0.0388
Well, moderate	24	17	
Poor	13	24	
EGFR mutation status			0.0486
Mutated (Exon 19, 20, 21)	10	20	
Wild type	27	21

**Table 3:** Multivariate analysis of OS for all patients

Variable	Hazard ratio	95% CI	*p *value
Age			
≤ 65	1		
> 65	1.22	0.60–2.49	0.58
Gender			
Female	1		
Male	0.63	0.28–1.41	0.27
Smoking status			
Nonsmoker	1		
Ever-smoker	0.92	0.44–1.93	0.82
Histologic type			
Non-Adeno	1		
Adenocarcinoma	0.83	0.341–2.02	0.68
Stage			
I–II	1		
III–IV	1.68	0.70–4.07	0.247
Differentiation			
Well, moderate	1		
Poor	2.16	1–4.69	0.05
EGFR mutation status			
Wide type	1		
Mutated	0.89	0.45–1.77	0.739
CAP1 level			
Low	1		
High	2	0.94–4.27	0.07

### CAP1 depletion inhibited proliferation and motility in lung cancer cell lines

**Fig. 5:**
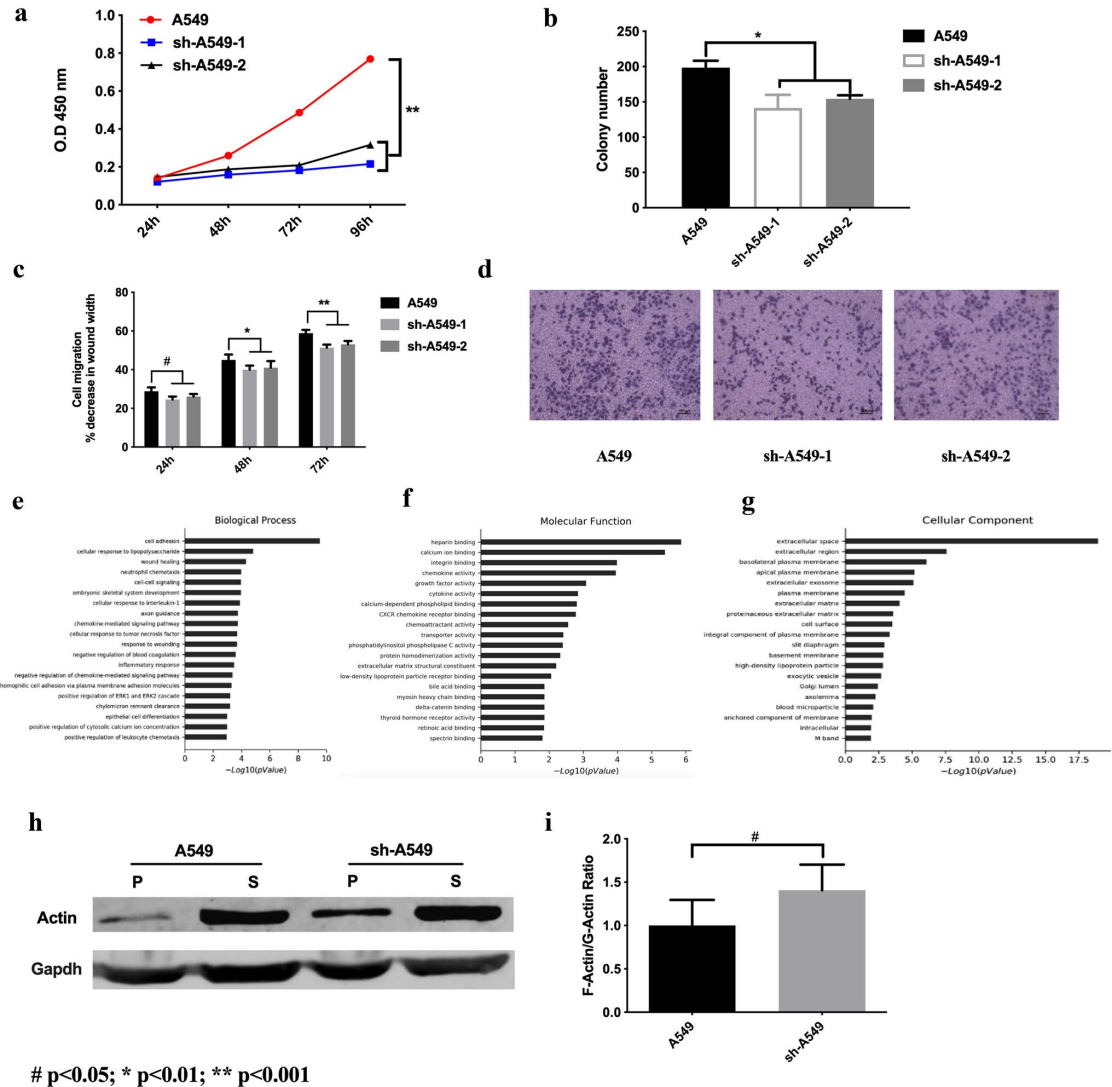
CAP1’s role in A549 cells. **a**, **b** CAP1 depletion inhibited proliferation in A549 cells validated by CCK-8 and Clone formation. **c**, **d** Wound-healing and transwell assays of CAP1 depletion in A549 cells. **e**–**g** The GO analysis of CAP1 knockdown A549 cells. **h**, **i** CAP1’s functions in regulating cell actin assembly (#*p* < 0.05; *p < 0.01; **p < 0.001)

To investigate the role of CAP1 in lung cancer cells, we transfected A549 cell line with lentivirus to stably knock down CAP1. Efficiency of CAP1 knockdown was detected by western blot, RT-PCR, and immunofluorescence (Fig.S1a, b). Knockdown of CAP1 exhibited an obvious impact on cells proliferation. Cell proliferation assays were performed. CCK-8 assay showed that CAP1 depletion inhibited proliferation in A549 cells (Fig. 5a, sh-1: *p* < 0.0001; sh-2: *p* < 0.0001). We performed clone formation assays to validate the results. The clone formation assay results were consistent with what we observed in CCK-8 assays; knockdown of CAP1 in A549 cells caused obvious reduction in the clone number (Fig. 5b, sh-1: *p* = 0.00925061; sh-2 *p* = 0.00233748). It was evident that CAP1 depletion inhibited proliferation in A549 cells. We evaluated the effect of CAP1 on metastasis of the A549 cells. Results from wound-healing assays revealed that the knocking down of CAP1 decreased migration ability of A549 cells (Fig. 5c) (sh-1 and sh-2: *p* < 0.0001). Furthermore, we performed transwell assays to validate the results (Fig. 5d), which were consistent with the previous wound-healing assays. These results showed that CAP1 depletion inhibited the motility in A549 cells. Lung cancer H1975 cells were used to validate the result that CAP1 depletion inhibited proliferation and motility. The efficacy of CAP1 knockdown by siRNA was detected by western blot and RT-PCR (Fig. S1c). CCK-8 (S1 and S2: *p* < 0.01) and clone formation assays (S1 and S2: *p* < 0.001) showed that after knockdown CAP1 in H1975, cells proliferation abilities were dramatically inhibited (Fig. S1d). Cell motility was examined by wound-healing (S1 and S2: *p* < 0.001) and transwell assays. The results showed that cell mobility were greatly inhibited after knockdown CAP1 (Fig. S1e, *p* < 0.001). To further understand the function of CAP1 in lung cancer cells, we extracted mRNA from A549 and sh-A549 cells for microarray analysis, and the analysis was performed by Shanghai OE Biotech Co., Ltd. The GO analysis revealed that CAP1 knockdown in A549 cells caused changes to the cell adhesion process, which impaired protein binding in molecular function. CAP1 knockdown also significantly changed the extracellular region in the cellular component (Fig. 5e–g).

### Role of CAP1 functions in regulating cell actin cytoskeleton

CAP1 is a type of actin-associated protein. We performed experiments to validate the effect of CAP1 depletion on the balance between F-actin and G-actin. We used high-speed centrifuge to obtain supernatant and pellet fraction to investigate CAP1’s impact on F-actin and G-actin. G-actin is the monomeric form of actin that can be polymerized into F-actin (Gunning et al. 2015). G-actin primarily exists in the supernatant, while F-actin mainly exists in the pellet (Kim et al. 2019). As shown in Fig. 5h, CAP1 knockdown A549 cells elevated actin in pellet, which meant that the F-actin/G-actin ratio was higher in CAP1 knockdown A549 cells. The loss of CAP1 leads to significant changes in the actin assembly in A549 cells, which was characterized by an increase in F-actin (Fig. 5i, *p* = 0.0486).

### Role of S308/S310 phosphorylation status in CAP1 functions in regulating lung cancer cells proliferation, migration, and invasiveness were examined in vitro

**Fig. 6:**
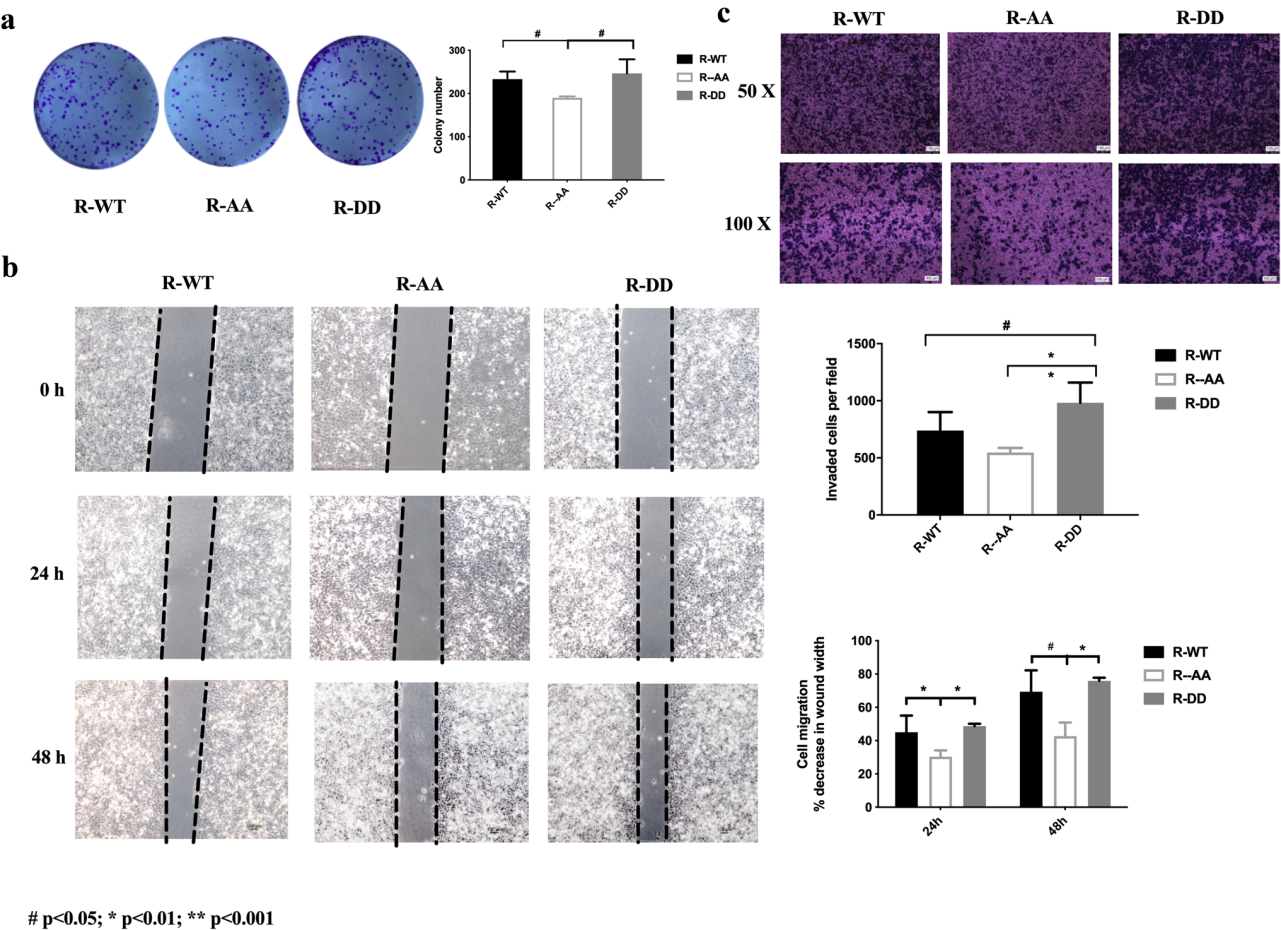
CAP1 S308/S310 phosphorylation promoted A549 cell proliferation and migration were examined in vitro. **a** Clone formation assay showed the R-AA group formed the least number of clones compared with R-WT and R-DD group. **b**, **c** The wound-healing and transwell assays showed that phosphorylation in both S308 and S310 in CAP1 stimulated the migration ability in A549 cells (#*p* < 0.05; *p < 0.01; **p < 0.001)

To investigate the impact of CAP1 phosphorylation status on proliferation, we separately transfected sh-A549 cells with the following specific phosphorylation state plasmids: CAP1 WT, AA, and DD. The CCK-8 and clone formation assays were performed, and the CCK-8 assay showed no significant difference between these groups. However, the clone formation assay showed that the AA group formed the least number of clones, which indicated that the de-phosphorylation in both S308 and S310 sites in CAP1 inhibited the proliferation ability in A549 cells (Fig. 6a) (WT vs. AA *p* = 0.0136222; AA vs. DD *p* = 0.0398847). To investigate the impact of CAP1 phosphorylation status on migration and invasiveness, we performed wound-healing and transwell assays by separately transfecting sh-A549 cells with the following specific phosphorylation state plasmids: CAP1 WT, AA, and DD. The wound-healing and transwell assays showed that both phosphorylated S308 and S310 sites in CAP1 stimulated the migration ability in A549 cells, while both de-phosphorylated S308 and S310 sites in CAP1 inhibited the migration ability in A549 cells. (Fig. 6b, c) (wound-healing WT vs. AA *p* = 0.0385446; AA vs. DD *p* = 0.00234934; Transwell WT vs. DD *p* = 0.0322082; AA vs. DD *p* = 0.000155958).

### Role of S308/S310 phosphorylation status in CAP1 functions in regulating lung cancer cells proliferation, migration, and invasiveness were examined in vivo

**Fig. 7:**
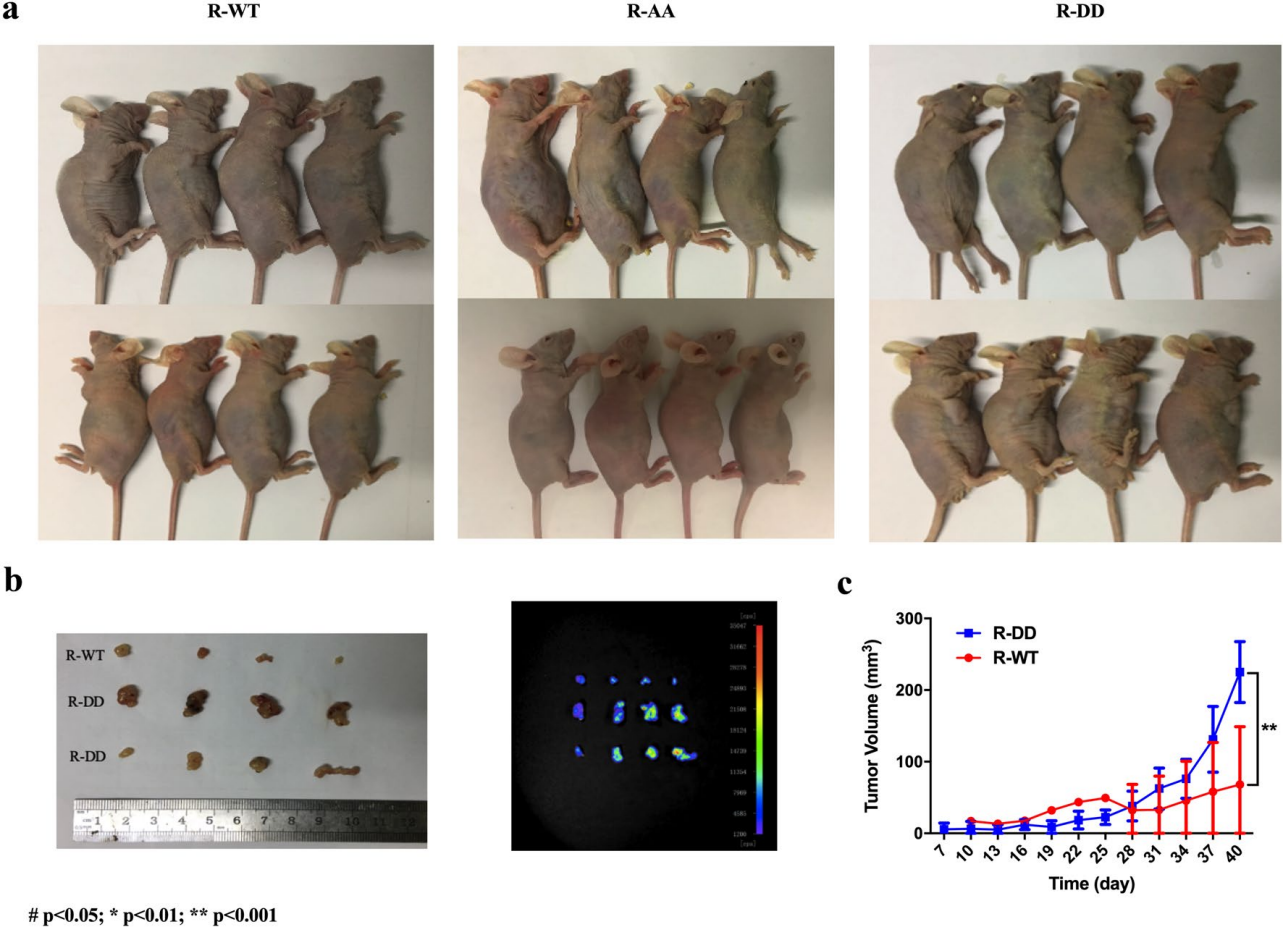
CAP1 S308/S310 phosphorylation promoted A549 cells proliferation was examined in vivo. **a** Re-expressed CAP1 WT, AA, and DD mutant sh-A549 cells were subcutaneously injected into nude mice. **b** Macroscopic view of xenografts with tumor in nude mice and viewed by small animal live imaging system. **c** Volumes of xenografts were shown (#*p* < 0.05; *p < 0.01; **p < 0.001)

**Fig. 8:**
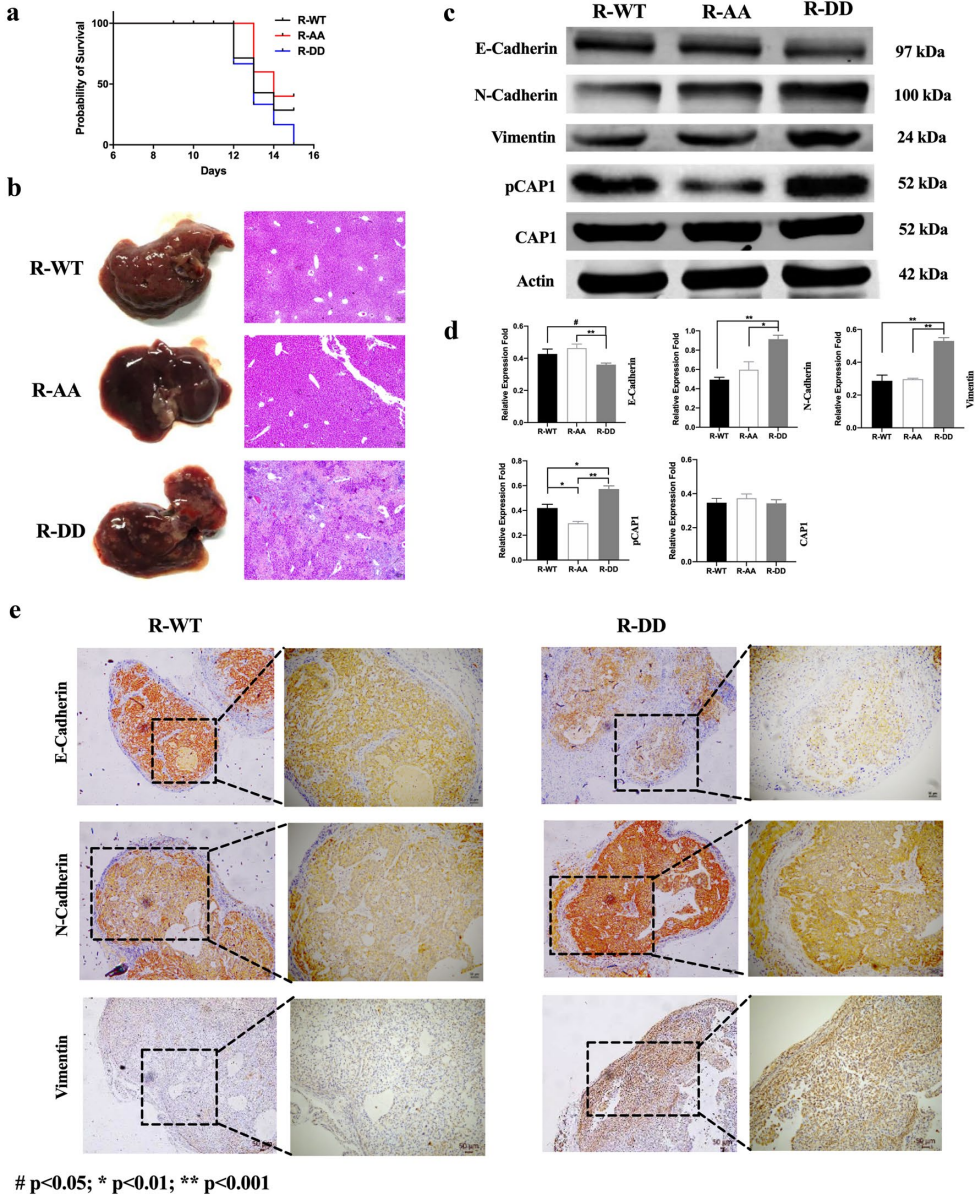
Phosphorylation of S308 and S310 in CAP1-reduced overall survival was examined in vivo and promoting EMT. **a** Kaplan–Meier survival curve of vein tail xenografts mice models. **b** Pathological biopsy of liver in vein tail xenografts models. **c**. **d** Western blot analysis for EMT-related proteins and quantified expression level of proteins. **e** Immunohistochemistry assessment of EMT-related proteins in subcutaneous xenografts (#*p* < 0.05; *p < 0.01; **p < 0.001)

We performed in vivo assays in nude mice by subcutaneously injecting sh-A549 cells transfected separately with WT, AA, and DD plasmids to verify the in vitro outcomes. The results showed that only the WT and DD groups formed tumors. Tumor formation rate was 0% in the AA group, and only 50% in the WT group compared to 100% in the DD group (Fig. 7a). Tumors in WT group grew slower and were smaller in size than that of the DD groups, which indicated that the phosphorylation of S308 and S310 in CAP1 promoted the proliferation ability in A549 cells (Fig. 7a–c) (R-WT 68.0751 ± 34.7477; R-DD 225.035 ± 34.747; *p* = 0.000155958). To verify the CAP1 phosphorylation impact on cells migration from the in vitro results, we injected sh-A549 cells transfected separately with WT, AA, and DD plasmids into nude mice by vein. The results showed that mice in the DD group had the lowest survival rate, and all mice died within 15 days after injection (Fig. 8a). We compared the survival rate of R-AA and R-DD, and determined a p value of 0.2903. Although the results did not show statistical significance, possibly due to limited sample size and survival time, there was a clear indication that the mice in R-DD group had the lowest survival rate. The mice were dissected and abnormalities were clearly visible in the livers of DD group’s mice (Fig. 8b). We performed H&E staining on the liver sections, and found that while there was a lot of necrosis, tumors were not present in the liver of mice in the DD group under the microscope. Compared with the livers in other groups, no pathological changes were found. These data strongly suggested that phosphorylation of CAP1 promoted lung cancer metastasis.

### Phosphorylation of S308 and S310 in CAP1 promoting EMT

EMT is a biological transformation process that allows polarized cells with epithelial phenotype to assume mesenchymal cell phenotype. To elucidate whether EMT is involved in pCAP1-mediated NSCLC migration, we examined the effects of CAP1 phosphorylated states on the levels of epithelial cell marker (E-cadherin) and mesenchymal cell markers (N-cadherin and Vimentin). Western blot analysis showed that after transfection with different phosphorylated states’ plasmids, the transcription level of N-cadherin and Vimentin was higher in the DD group. However, E-cadherin was lower in the DD group compared with the WT and AA groups (Fig. 8c.d). We also observed the levels of EMT-related markers in subcutaneous tumors of nude mice by immunohistochemistry, which showed that the levels of E-cadherin were decreased following CAP1 phosphorylation, with an increased expression of N-cadherin and Vimentin (Fig. 8e). Overall, these results indicated that phosphorylation CAP1 could induce the migration capabilities of human lung cancer cells through promoting EMT.

## Discussion

Lung cancer is a major threat to human health due to the high morbidity and mortality rates (Denisenko et al. 2018; Zhong et al. 2019). Lung cancer has different subtypes, in which non-small cell lung cancer (NSCLC) comprises 85% of all lung cancer (Uprety et al. 2020). NSCLC has been increasingly recognized as a molecularly and genetically heterogeneous disease, and the metastasis is the primary reason for treatment failure and often results in patient death (Arbour and Riely 2019). However, the mechanism of lung cancer metastasis remains unclear.

Human CAP1 gene is present on chromosome 1. The protein encoded by this gene, also named CAP1, contains 475 amino acids. CAP1, an actin-binding protein, plays an important role in cell movement and regulates cell morphology (Zhou et al. 2014). Our previous study showed that the expression of CAP1 differs in different metastatic lung cancer tissues, which indicates that CAP1 is a cancer-related protein (Tan et al. 2013). CAP1 is a type of protein that can be phosphorylated. The two phosphorylated sites S308 and S310 in human CAP1 correspond to the phosphorylated sites S307 and S309 in mice, respectively (Zhang et al. 2016).

Post-translational modifications (PTMs) are a series of chemical synthesis after protein translation and are an important way to regulate protein function. PTMs participate in many biological processes, including both physiological and pathological states (Liu et al. 2016). PTMs are available in a variety of ways, including acetylation, methylation, glycosylation, phosphorylation, etc. (Czuba et al. 2018). Phosphorylation is a common, but important way of PTMs. It is involved in the regulation of multiple life activities. Protein phosphorylation mainly occurs in two amino acids: one is serine (including threonine) and the other one is tyrosine (Silva et al. 2013). Phosphorylation of these amino acids can produce different effects. After serine phosphorylation, the protein is activated by changing the spatial structure. However, in tyrosine phosphorylation, in addition to activating the protein, the activated protein can also connect with other proteins to form a complex (El Turk et al. 2018). Phosphorylation or de-phosphorylation regulates intracellular signal transduction, gene expression, cell cycle, and many other cellular processes, including processes in cancer (Martire et al. 2019).

The purpose of our study was to observe the role of CAP1 in lung cancer and its PTM on lung cancer cells’ proliferation and metastasis. We applied extensive bioinformatics analysis to determine CAP1’s role in different cancers and especially in lung cancer. The mRNA level of CAP1 was overexpressed in most types of cancer tissues when compared with normal tissues. CAP1 high expression correlated with shorter OS in various cancers which includes lung cancer and shorter DFS in lung cancer, as well. CAP1 higher transcription level was observed in TP53-mutant lung cancer patients. Protein CAP1 phosphorylation rate was higher in lung cancer tissue compared with normal tissue and had a direct correlation to the disease stage and grade. TCGA database included in cBioPortal (Hoadley et al. 2018) showed CAP1 gene had a high correlation with some key cancer-related genes which further confirmed the carcinogenesis role of CAP1. According to biological process (GO) analysis, CAP1 and its PPI network mainly played a role in actin filament organization, cell morphogenesis regulation, and response to stimulus. Its molecular function mainly focused on actin or other protein binding. As for the cellular component, CAP1 mainly existed in cytoplasm and extracellular region. We collected serum from NSCLC patients and normal people for ELISA experiment. The results showed that the translational level of CAP1 was significantly upregulated in NSCLC patients compared with normal people, and poor differentiation and EGFR mutation status were associated with high serum CAP1 protein level. Higher serum CAP1 protein level correlated with poor prognosis, and higher pCAP1 and CAP1 protein levels were observed in lung cancer patients’ tumor tissue compared with adjacent normal tissue. Then, we investigated the role of CAP1 in A549 cells and found that CAP1 knockdown prohibited A549 cells proliferation and metastasis. It is well known that A549 cells harbored KRAS mutant. KRAS mutation is more commonly found in western countries in about 20–25% of the population, while it is only present in 10–15% of the population in Asian countries. The KRAS mutation is highly detected in heavy-smoking patients, most of them with adenocarcinoma (Chapman et al. 2016). KRAS mutation is the downstream signal pathway of the EGFR mutation (Midha et al. 2015). EGFR mutation is the main driver of NSCLC. The mutation rate in Asian population is as high as 40–50%, while in Western population, the rate is 10–20% (Guan et al. 2016; Li et al. 2017). Some studies showed that patients who harbored EGFR mutation were more likely to develop brain metastasis (Ge et al. 2017; Page et al. 2020). The underlying mechanism of cancer development and gene mutation is still not well understood. From our research, we collected 78 lung cancer serum samples; among them, 30 are EGFR mutation samples; it is hard to explore the relationship between EGFR mutation and CAP1 expression with limited sample size. Expanding EGFR mutation samples size will be our next step to get deeper understanding of this relationship. CAP1’s function was validated in EGFR mutant H1975 cells, and it functions as an oncogene both in A549 and H1975 cells. We investigated the underlying mechanisms, and the previous research showed CAP1 is a type of actin-binding protein, which impacts cell motility by affecting actin assembly (Zhou et al. 2014). G-actin is the monomeric form of actin that can be polymerized into F-actin. We compared the ratio of F-actin/G-actin between A549 and sh-A549 cells. Our experiments showed that the loss of CAP1 led to increased F-actin in A549 cells. Thus, our results provided more evidence to validate the connection between CAP1 and actin and CAP1’s role in regulating actin assembly.

Previous study showed that S308 and S310 of human CAP1 protein could be phosphorylated and could act in tandem (Zhang et al. 2016). We separately transfected sh-A549 cells with specific phosphorylated mutant plasmids (WT, AA, and DD) and observed the effects of the phosphorylation mutations. We did not get a positive result in the CCK-8 experiment, but in the clone formation experiment, we found that de-phosphorylated S308 and S310 sites in CAP1 inhibited the proliferation ability in A549 cells. Then, we performed wound-healing and transwell assays and discovered that phosphorylated S308 and S310 sites in CAP1 stimulated the migration ability in A549 cells. We also performed in vivo experiments to verify the in vitro results. By subcutaneously injecting nude mice with transfected specific mutant plasmids A549 cells, the processes of proliferation were mimicked. We found that the DD group formed more subcutaneous tumors and with larger tumor sizes than the other groups. We also injected cells transfected with specific mutant plasmids into the nude mice by vein. Mice in DD group showed shortest survival time compared with WT and AA group. Pathological biopsy showed that only the nude mice in DD group exhibited necrosis in the liver. These outcomes strongly indicated that the phosphorylation of CAP1 plays a prominent role in tumor development and acceleration of lung cancer metastasis.

From the above results, we concluded that CAP1 can act as an oncogene in lung cancer. CAP1 phosphorylation promoted proliferation and metastasis in A549 cells, and de-phosphorylation of CAP1 showed the opposite effects. We also observed the impacts of CAP1 phosphorylation and found that CAP1 phosphorylation promoted EMT. EMT refers to the biological process by which epithelial cells are transformed into mesenchymal phenotypes (Yuan et al. 2020). EMT plays an important role in the process of cancer metastasis. Tumor cells gained a stronger ability to move through the epithelial–mesenchymal transition process, resulting in metastasis (Lee et al. 2017). A large number of studies have found that during this process, the expression of the characteristic epithelial adhesion protein, E-Cadherin is decreased, while the expressions of the mesenchymal markers, N-Cadherin and Vimentin, are increased (Ding et al. 2018). Through this transformation, epithelial cells acquire a stronger migration and invasion ability (Sesumi et al. 2017). Our experiments showed that the expression of E-Cadherin was decreased in the DD group cells, while the expressions of the mesenchymal markers, N-Cadherin and Vimentin, were increased. In contrast, the expression of E-cadherin was increased in the AA group cells, while the expressions of the mesenchymal markers, N-Cadherin and Vimentin, were reduced. We also observed the expression of EMT markers in subcutaneous tumors in animal models. The mesenchymal markers in the DD group were higher than those in the WT group. Thus, we verified that CAP1 phosphorylation promotes the development of lung cancer by promoting epithelial–mesenchymal transition, both in vitro and in vivo.

## Conclusions

Taken together, our research showed that CAP1 was overexpressed in most cancers, including lung cancer. CAP1 high transcription level associated with shorter OS in various cancers. In lung cancer, high CAP1 mRNA level was observed with a shorter OS and DFS. CAP1 showed a higher phosphorylation rate in lung cancer, and the phosphorylation rate had a direct correlation to the stage and grade of lung cancer. Gene CAP1 was associated with many cancer key genes, and GO analysis showed that protein CAP1 has a connection with actin. CAP1 was overexpressed in NSCLC serum samples and correlated with poor clinical outcomes. Knockdown of CAP1 in A549 and H1975 cells inhibited proliferation and migration. It also led to an increase in the ratio of F-actin/G-actin in A549 cells. Phosphorylated S308 and S310 in CAP1 promoted the proliferation and migration of lung cancer cells. When de-phosphorylated, these two sites in CAP1 showed the opposite effects. We validated these results both in vitro and in vivo. CAP1 phosphorylation promoted migration via EMT was validated. These findings indicated that CAP1 phosphorylation might be a novel target for lung cancer treatment.

## Supplementary Information

Below is the link to the electronic supplementary material.

Supplementary file1 Figure S1 CAP1 was knockdown in A549 cells and H1975 cells. (TIFF 2745 KB)

## Abbreviations

CAP1: Adenylate cyclase-associated protein 1
pCAP1: Phosphorylated adenylate cyclase-associated protein 1
NSCLC: Non-small cell lung cancer
GFP: Green fluorescent protein
CCK-8: Cell Counting Kit-8
RT-PCR: Real-time quantitative PCR
GAPDH: Glyceraldehyde-3-phosphate dehydrogenase
FBS: Fetal bovine serum
PBS: Phosphate buffer saline
PVDF: Polyvinylidene fluoride
EMT: Epithelial–mesenchymal transition
PTM: Post-translational modification
OS: Overall survival
DFS: Disease-free survival
GO: Gene Oncology
