# Supplementary figures and images for: Phosphorylation of CAP1 regulates lung cancer proliferation, migration, and invasion
Source: J Cancer Res Clin Oncol. 2021 Oct 12;148(1):137–53. doi: 10.1007/s00432-021-03819-9 (PMC8752530; doi:10.1007/s00432-021-03819-9)

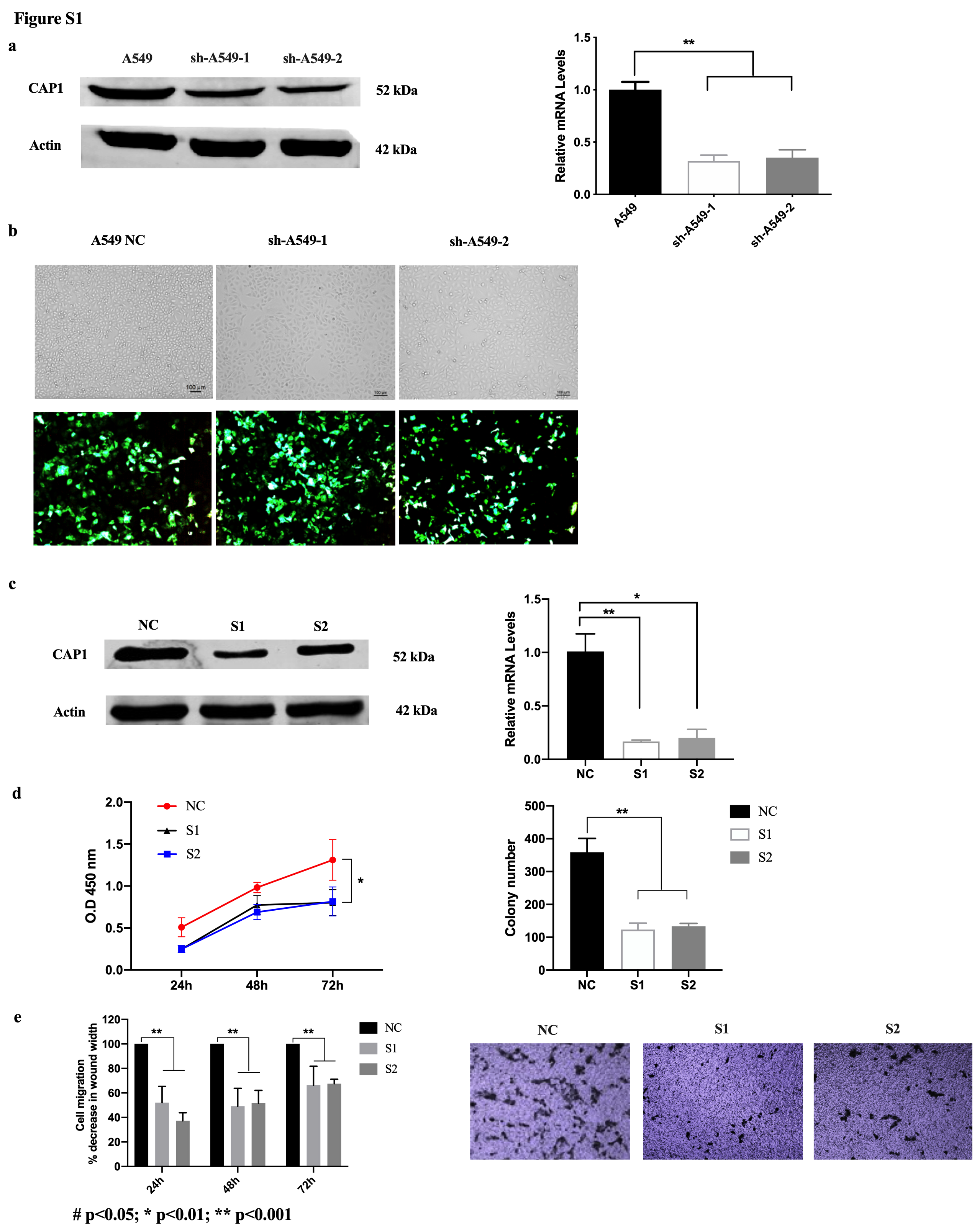

Supplement: Supplementary file 1 — Supplementary file1 Figure S1 CAP1 was knockdown in A549 cells and H1975 cells. (TIFF 2745 KB) [file 432_2021_3819_MOESM1_ESM.tiff]
